# Exploratory analysis of obsessive compulsive symptom dimensions in children and adolescents: a Prospective follow-up study

**DOI:** 10.1186/1471-244X-6-1

**Published:** 2006-01-05

**Authors:** Richard Delorme, Arnaud Bille, Catalina Betancur, Flavie Mathieu, Nadia Chabane, Marie Christine Mouren-Simeoni, Marion Leboyer

**Affiliations:** 1 INSERM U513, Faculté de Médecine, Université Paris XII, Créteil, France; 2 Service de Psychopathologie de l'Enfant et de l'Adolescent, Hôpital Robert Debré, Assistance Publique-Hôpitaux de Paris, Paris, France; 3 Service de Psychiatrie, Hôpital Henri Mondor et Albert Chenevier, Assistance Publique-Hôpitaux de Paris, Créteil, France

## Abstract

**Background:**

Recent statistical approaches based on factor analysis of obsessive compulsive (OC) symptoms in adult patients have identified dimensions that seem more effective in symptom-based taxonomies and appear to be more stable over time. Although a phenotypic continuum from childhood to adulthood has been hypothesized, no factor analytic studies have been performed in juvenile patients, and the stability of OC dimensions in children and adolescents has not been assessed.

**Methods:**

This study was designed to perform an exploratory factor analysis of OC symptoms in a sample of children and adolescents with OC disorder (OCD) and to investigate the course of factors over time (mean follow-up period: four years).

**Results:**

We report for the first time that four symptom dimensions, remarkably similar to those previously described in adults, underlined the heterogeneity of OC symptoms in children and adolescents. Moreover, after follow-up, the symptom dimensions identified remained essentially unmodified. The changes observed concerned the intensity of dimensions rather than shifts from one dimension to another.

**Conclusion:**

These findings reinforce the hypothesis of a phenotypic continuum of OC symptoms from childhood to adulthood. They also strengthen the interest for investigating the clinical, neurobiological and genetic heterogeneity of OCD using a dimension-based approach.

## Background

Obsessive-compulsive disorder (OCD) is a severe, chronic neuropsychiatric illness affecting 1%–3% of children and adolescents, characterized by recurrent, distressing, unwanted thoughts (obsessions) and repetitive, ritualistic behaviors (compulsions). Although standard nomenclatures regard OCD as a unitary nosological entity, patients typically display a wide variety of obsessions and/or compulsions of varying severity. The diversity of demographic and clinical characteristics [1,2], nature of OCD symptoms (predominance of obsessions or compulsions), associated comorbid disorders [3], and response to treatment interventions [4], suggests that important subtypes of OCD may exist. However, earlier attempts for symptom-based taxonomies have met limited success. Indeed, the categorical studies that used mutually exclusive subgroups of patients (e.g., checkers versus washers) to determine a specific relationship with clinical variables such as psychopathology, response to treatment, or genetic and neurobiologic variables were relatively uninformative, and follow-up studies reported important changes in the constellation of OC symptoms over time. In the longest follow-up study ever published, covering a period of 40 years from baseline to re-evaluation, Skoog and Skoog [5] reported that more than half of the adult OCD patients had a qualitative change in their symptom themes. In addition, a study in children and adolescents reported drastic changes of OC symptoms over time [6], since none of 79 juvenile patients maintained the same pattern of symptoms during the follow-up period lasting from 2 to 16 years. These two studies, which used a categorical approach [Skoog and Skoog used an idiosyncratic definition of symptom categories, whereas Rettew et al. used the Yale-Brown Obsessive Compulsive Scale (Y-BOCS) symptom checklist], concluded that OC symptoms displayed a marked instability across time.

In contrast to these findings, more recent statistical approaches, based on factor analysis and taking into account the complexity of OC symptoms seen in patients, tended to identify dimensions that could be more effective in symptom-based taxonomies, and preliminary findings suggest that they could be more stable over time (for review, see [7]). These studies suggest the presence of certain broad symptom domains that capture the heterogeneity of OC symptoms. Four major dimensions accounting for more than 60% of the variance have been consistently identified: the first dimension is characterized by symmetry and ordering obsessions and compulsions; the second is composed of aggressive, religious, sexual, and somatic obsessions, and checking compulsions; the third includes contamination obsessions and cleaning/washing compulsions; and the fourth comprises hoarding obsessions and compulsions [8-12]. The validity of the four-factor model has gained support from studies showing that these factors: i) are characterized by specific clinical features such as sex, age at onset, comorbid tics and personality disorders [8,10,13,14]; ii) are mediated by relatively distinct patterns of activation of fronto-striato-thalamic circuits [15]; iii) represent predictors of the response to serotonin reuptake inhibitors [11]; and finally iv) are related to different patterns of genetic transmission [16], and could be associated with specific susceptibility loci [17].

To date, only one study has investigated the longitudinal stability of this four factor structure. A preliminary prospective study in adult OCD patients reported that symptom dimensions appeared to be more stable over time in adults than previously suggested [18]. Although there was a decrease in the intensity of dimensions, not correlated to the overall reduction of the severity of the disorder, shifts from one dimension to another were rare at six months intervals up to two years post-initial assessment. No factor analytic studies have been performed in child and adolescent populations. The only study performed to date about symptom stability in pediatric OCD was the study of Rettew et al. [6] mentioned previously, which reported a change of symptoms over time. These findings could be due to the statistical methodology of the study, based on symptom categories rather than symptom dimensions.

In order to explore the multidimensionality of OC symptoms in pediatric patients and the temporal stability of the dimensions, we performed a factor analysis of OC symptoms in a sample of children and adolescents with OCD and investigated the course of these factors after a mean follow-up period of 3.8 ± 0.9 years. Assuming a developmental continuity of OCD over time [19,20], we hypothesized that exploratory factor analysis in a sample of children and adolescents with OCD should produce similar dimensions to those reported in adult populations, and as a consequence, should show temporal stability.

## Methods

### Participants

Patients seeking treatment at a university-based OCD clinic and meeting DSM-IV criteria for OCD were recruited between 1998 and 2001 to participate in a genetic study. Seventy-three patients (44 males and 29 females), aged 7 to 18 years at interview (mean ± SD: 13.7 ± 2.7 years) were included in the present study. All subjects were from Caucasian descent. Their mean (± SD) age at onset of OCD was 9.5 ± 3.2 (range 3 to 17) years. At inclusion, 59 of them (70.8%) had been receiving serotonergic receptor inhibitors for 12 weeks or more, and 23 (31.5%) had received or were receiving a trial of behavior therapy. Their mean (± SD) scores on the Y-BOCS were 22.2 ± 6.0 for total score, 10.8 ± 3.2 for the obsession subscale, and 11.3 ± 3.5 for the compulsion subscale.

All subjects were contacted for a follow-up evaluation, and of the initial group of patients, 42 (57.5%) participated in the re-evaluation, 3.8 ± 0.9 years (mean ± SD) after baseline. Data comparability analyses showed no differences among those who participated or not in terms of sex distribution, age, age at onset and Y-BOCS total score at baseline. In the re-evaluated group, a significant decrease of the mean (± SD) total score of the Y-BOCS from baseline (22.3 ± 5.3) to follow-up (17.1 ± 5.7) was observed (Student's paired t-test, two-tailed, t = 4.64, df = 41, p < 0.001).

The proband's consent and written parental consent were obtained after the purpose of the study and the nature of the interviews were described in age-appropriate language. The clinicians who conducted the interviews were blind to the hypothesis. The Research Ethics Board of the Pitié-Salpêtrière hospital reviewed and approved the study (CCPPRB12-05).

### Clinical measures

At baseline, lifetime psychiatric evaluation was carried out during a direct interview by trained psychiatrists using the Kiddie Schedule for Affective Disorders and Schizophrenia – Epidemiologic version [21]. Patients with comorbid diagnoses were not excluded from the study, provided that OCD was the dominant disorder for which treatment was sought. The collection of information concerning symptoms associated with OCD was done in a two-stage process. For the initial stage, the family self-report questionnaire designed by the Tourette Syndrome Association Genetic Consortium [1995 version], and based on the tic inventory and ordinal severity scales of the Yale Global Tic Severity Scale [22] and on the symptom checklist and ordinal scales of the Y-BOCS, was used [23]. Earlier versions of these instruments have been shown to have good agreement with expert clinicians rating of tic and OC symptom severity [24]. We decided to use the adult version of the Y-BOCS checklist instead of the children's version, as Rettew et al. 1992 [6] did to explore symptom stability in pediatric OCD. The children and the adult versions of the YBOCS are very similar: the ordinal scales of both versions are the same, and the seven major obsession categories and the six major compulsion categories are also the same. Only the miscellaneous obsessions and compulsions differ in the two versions, and are subdivided in the children version in miscellaneous, magical thinking/superstitions and excessive games categories. However, miscellaneous categories were not included in the factor analysis.

In a second stage, an experienced clinician reviewed these symptom ratings with each patient and their parents to ensure their accuracy and validity. At follow-up, performed 3.8 ± 0.9 years after the initial evaluation, the family self-report questionnaire and the Y-BOCS scores were again filled-in with the help of a skilled psychiatrist.

### Data analysis

The exploratory analysis of symptom dimensions was performed following the methods used in adults [10]. A principal component factor analysis with a varimax rotation was carried out on symptom counts from the 13 a priori categories in the Y-BOCS symptom checklist filled-in by patients at baseline. We summed the number of symptoms present during the initial evaluation for each category in order to take maximum advantage of the variance within each of the data sets. The patients' scores on the symptom dimensions were computed by multiplying the respective category-specific coefficients by the number of items endorsed for each of the 13 symptom categories by each participant.

At follow-up, the stability of OC symptoms was explored using the methods described by Mataix-Cols et al. [18]. First, we estimated for each of the 13 Y-BOCS symptom categories the probability that symptoms changed or remained unchanged between baseline and follow-up using the McNemar's test. Second, we explored the stability of OCD symptom dimensions. Patients' scores on the symptom dimensions were obtained by multiplying the respective category-specific coefficients identified at baseline by the number of items endorsed for each of the 13 symptom categories by each participant during the second evaluation. We then performed a series of stepwise multiple regression models in which each symptom dimension at follow-up was the dependent variable and all four OCD dimensions at baseline were entered as independent variables. All statistical analyses were performed with the aid of SPSS/PC+ computer software.

## Results

### Symptom categories of obsessions and compulsions at baseline and follow-up

Frequencies of the 13 major symptom categories (seven categories of obsessions and six categories of compulsions) of the Yale-Brown checklist at baseline and follow-up are shown in Table 1. Most of our patients maintained their symptoms across follow-up. However, we observed a slight but significant tendency for change during follow-up in religious and somatic obsessions and in repeating compulsions. In addition, about one third of the patients showed modifications in aggressive and contamination obsessions and in cleaning, counting and ordering compulsions across time, albeit not statistically significant. A large majority of patients reported symptoms that spanned between the different categories. Specifically, at baseline, patients had symptoms belonging to 2.6 ± 2.1 categories of obsessions (among seven) and to 2.2 ± 1.8 categories of compulsions (among six). At follow-up, we observed a significant decrease in the number of categories of obsessions (1.9 ± 2.0), but not in the number of categories of compulsions (1.8 ± 1.7) (Wilcoxon two sample test, z = -2.43, p = 0.02 and z = -0.75, p = 0.45, respectively). Since a significant decrease in the severity of OCD was observed during follow-up, analyses with the percent drop of the total Y-BOCS over the follow-up period as a co-variable were performed. They yielded non significant coefficients.

**Table 1:** Symptoms categories at baseline and at follow-up among children and adolescents with OCD

Symptom category in the Yale-BOCS	Symptoms present at baseline						Symptoms present at follow-up (n = 42)			Patients whose symptoms changed (n = 42)
					
	Entire sample (n = 73)			Reevaluated sample (n = 42)						
	n (%)	mean (SD)^a^	median (IQR)^a^	n (%)	mean (SD)^a^	median (IQR)^a^	n (%)	mean (SD)^a^	median (IQR)^a^	n (%)
Obsessions										
Aggressive	35 (47.9)	2.3 (1.6)	2 (2)	21 (50.0)	2.1 (1.3)	2 (2)	14 (33.3)	1.8 (1.4)	1 (1)	17 (40.5)
Contamination	33 (45.2)	2.6 (1.4)	2 (2)	17 (40.5)	2.1 (1.3)	2 (2)	14 (33.3)	1.4 (0.6)	1 (1)	15 (35.7)
Sexual^b^	10 (13.7)			4 (9.5)			5 (11.9)			5 (11.9)
Hoarding^b^	15 (20.5)			9 (21.4)			8 (19.0)			11 (26.2)
Religious	34 (46.6)	1.3 (0.5)	1 (1)	20 (47.6)	1.2 (0.4)	1 (0)	11 (26.2)	1.2 (0.4)	1 (0)	19 (45.2)^c^
Symmetry	28 (38.4)	1.7 (0.9)		17 (40.5)	1.7 (0.9)	1 (2)	13 (31.0)	1.7 (1.0)	1 (2)	12 (28.6)
Somatic	34 (46.6)	1.3 (0.5)	1 (1)	22 (52.3)	1.3 (0.5)	1 (1)	14 (33.3)	1.2 (0.4)	1 (0)	14 (33.3)^c^
Compulsions										
Cleaning	31 (42.5)	1.6 (0.8)	1 (1)	16 (38.1)	1.7 (0.8)	1 (1)	11 (26.2)	1.6 (0.5)	1 (1)	11 (26.2)
Checking	36 (49.3)	1.9 (1.0)	2 (1)	18 (42.9)	1.8 (0.9)	2 (1)	13 (31.0)	1.8 (1.3)	1 (1)	9 (21.4)
Repeating	35 (47.9)	1.5 (0.5)	2 (1)	21 (50.0)	0.8 (0.9)	2 (1)	14 (33.3)	1.4 (0.5)	1 (1)	11 (26.2)^c^
Counting^b^	20 (27.4)			12 (28.6)			11 (26.2)			13 (31.0)
Ordering	28 (38.4)	1.7 (0.8)	2 (1)	15 (35.7)	1.7 (0.8)	2 (1)	20 (47.6)	1.6 (0.5)	2 (1)	13 (31.0)
Hoarding^b^	12 (16.4)			6 (15.0)			5 (11.9)			7 (16.7)

^a ^Statistics were calculated in subjects reporting the symptom

^b ^These categories contain only one item

^c ^Tendency for a significant change (McNemar's test, df = 1, p = 0.06)

SD: standard deviation; IQR: interquartile range

### Exploratory factor-analysis of symptom dimensions at baseline

The principal-component factor analysis based on symptom categories suggested the presence of four factors with eigenvalues > 1.0, accounting for more than 64% of the total variance (Table 2). The first factor, which was named symmetry/ordering dimension, represented 35% of the total variance and included symmetry obsessions and ordering, repeating and checking compulsions, with loadings ranging from 0.61 to 0.83. The second factor, which reflected an obsessive dimension, represented 13% of the variance and was composed of aggressive, sexual and somatic obsessions, associated with counting compulsions; all loadings ranged from 0.54 to 0.86. The third factor, named contamination/cleaning dimension, accounted for 11% of the variance and was composed of contamination and religious obsessions, associated with cleaning compulsions, with loadings ranging from 0.63 to 0.84. The fourth and smallest factor, which was named hoarding dimension, accounted for 8% of the variance and included hoarding obsessions and compulsions, with a loading of 0.87 and 0.73, respectively.

**Table 2:** Varimax rotated factor structure for Yale-Brown Obsessive Compulsive Scale Symptom Checklist Category Scores obtained in 73 OCD patients evaluated at baseline^a^

	Factor Loading			
	
	Factor 1 Symmetry/Ordering	Factor 2 Obsessive	Factor 3 Contamination/Cleaning	Factor 4 Hoarding
**Symptoms categories**				
Obsessions				
Aggressive	0.30	**0.70**	0.18	0.20
Contamination	-0.02	0.27	**0.84**	-0.02
Sexual	0.03	**0.86**	0.15	0.03
Hoarding	0.12	0.21	0.02	**0.87**
Religious	0.20	0.29	**0.63**	0.15
Symmetry	**0.80**	0.02	0.17	0.22
Somatic	-0.05	**0.54**	0.38	0.37
Compulsions				
Cleaning	0.36	-0.24	**0.69**	0.07
Checking	**0.61**	0.31	0.38	-0.03
Repeating	**0.62**	0.56	-0.16	0.03
Counting	0.49	**0.56**	-0.16	0.03
Ordering	**0.83**	0.17	-0.06	0.25
Hoarding	0.40	-0.08	0.11	**0.73**
				
**Statistic**				
Eigenvalue	4.58	1.65	1.42	1.05
Variance explained	35.2%	12.7%	11.0%	8.10%

^a ^Based on the assumption that the scores on the 13 symptoms categories are normally distributed

### Stability of symptom dimensions from baseline to follow-up

**Figure 1:**
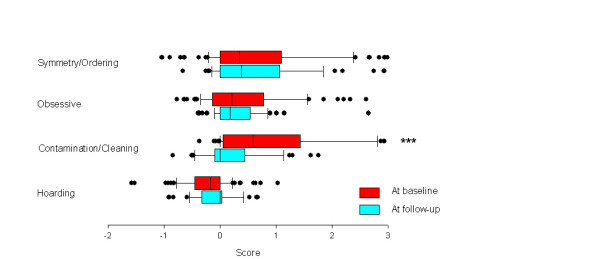
Symptom dimensions at baseline and at follow-up among 73 children and adolescents with OCD. Data represent the center, spread and skew of the distribution. *** p < 0.001 (Student's paired t-test, two-tailed, t = 4.80, df = 41).

The symmetry/ordering dimension, the obsessive dimension and the hoarding dimension remained unchanged across time (Student's paired t-tests, two-tailed, t = 0.29, df = 41, p = 0.77; t = 0.90, df = 41, p = 0.37; t = -0.45, df = 41, p = 0.66, respectively). By contrast, the contamination/cleaning dimension was significantly modified between baseline and follow-up (Student's paired t-test, two-tailed, t = 4.80, df = 41, p < 0.001) (Figure 1). Analysis with the percent drop of the total Y-BOCS over the follow-up period as a co-variable yielded a non significant coefficient. Multiple regression analyses showed strong partial correlations between each dimension at baseline and at the time of re-evaluation, except for the hoarding dimension (Table 3). Cross-dimensional correlations were rare and limited to the contamination/cleaning dimension, which showed small and negative correlations with the hoarding and symmetry/ordering dimensions.

**Table 3:** Partial correlations of OCD symptom dimensions at baseline and follow-up, determined by multiple regression analyses^a^

	**OCD dimensions at follow-up**			
	
	Symmetry/Ordering	Obsessive	Contamination/Cleaning	Hoarding
	
**OCD dimensions at baseline**				
Symmetry/Ordering	0.59^b^	0.07	-0.36^d^	-0.19
Obsessive	0.09	0.39^c^	0.22	-0.25
Contamination/Cleaning	0.05	-0.04	0.58^b^	-0.22
Hoarding	0.06	-0.11	-0.33^d^	0.24

^a ^R^2 ^ranged from 0.15 to 0.50; ^b ^p < 0.001; ^c ^p < 0.01; ^d ^p < 0.05

## Discussion

This study was designed to perform an exploratory factor analysis of OC symptoms in a sample of children and adolescents with OCD and to investigate the course of factors after a period of four years in average. We report for the first time that four symptom dimensions, remarkably similar to those previously described in adults, underlined the heterogeneity of OC symptoms in children and adolescents. Moreover, after follow-up, the symptom dimensions identified remained essentially unchanged. The changes concerned mainly the intensity of dimensions rather than shifts from one dimension to another, and were not correlated with the overall reduction in OCD severity over time.

### Symptom dimensions in children and adults

The four factors solution that emerged and best explained the phenotypic variance in our sample of juvenile OCD patients shows a high degree of consistency with the five large exploratory studies previously performed in adult samples [8-12]. The multidimensionality of OC symptoms was also supported by a confirmatory study performed in a large sample of OCD adult patients [25] that tested different models of symptom groupings and found that only the model composed of four factors fitted adequately.

The first factor that we observed, regrouping symmetry obsessions and ordering, repeating and checking compulsions, was also reported in adults. The symmetry obsessions and ordering and repeating compulsions have been reported to be associated and constitute a single dimension in the majority of studies performed in adults. In contrast, the presence of checking compulsions in this dimension is somewhat surprising. However, it has been recently suggested that the checking category could be a heterogeneous entity, i.e., that some symptoms could be associated with one dimension and the others with other dimensions [25]. Indeed, in a confirmatory analysis, Summerfeldt et al. [25] observed that the loadings of checking compulsion items on the predicted factor one spanned from 0.19 to 0.66. This could explain discrepancies between studies in which checking was found to be associated with contamination and cleaning symptoms [8], with aggressive, sexual, and religious obsessions [10], or with symmetry, ordering and repeating symptoms in our study. Thus, the integration of the checking category in a specific dimension could depend on clinical characteristics that are not directly related to the theme of the associated obsessions and/or compulsions. Specifically, the presence of comorbid chronic tics or Tourette's syndrome could influence the integration of checking items in a specific dimension. Leckman et al. [10] reported that OCD patients with comorbid chronic tic disorder or Tourette's syndrome had high loading on both obsessions and checking as well as symmetry and ordering factors. Given that 50% of our patients had a comorbid chronic disorder [26], this could explain why checking was associated with symmetry, ordering, and repeating symptoms.

The second factor that we obtained was composed essentially of aggressive, sexual and somatic obsessions. A similar dimension was also reported in studies performed in adults and seems to correspond to the pure obsessions factor described by Baer [8], and to the obsessive component of the aggressive/checking factor obtained by Leckman et al. [10], Summerfeldt et al. [25], and Cavalini et al. [9]. However, in our sample of juvenile patients, religious symptoms were not included in this 'obsessive factor' but in the contamination/cleaning factor. Discrepancies between our results and studies performed in adults could be specifically explained by differences in the chronological age of the patients enrolled in the studies. The emergence of religious obsessions is known to depend on the age of patients. Children have less frequent religious worries than adolescents [27] or adults [28]. Nevertheless, the relationship between religious obsessions and the contamination/cleaning factor observed in our study had been suggested previously. A strong correlation (r = 0.63) between the contamination/cleaning factor and the aggressive/checking factor, which contains the religious category, was reported in adults [29].

The symptomatic structure of the third and the fourth factors that we obtained in our child and adolescent population, were consistent with factors described in all studies performed in adults (with the exception of the religious obsessions discussed above). The hoarding dimension, in particular, has been reported to be associated with increased psychiatric comorbidity, as well as with poor response to serotonergic inhibitors and to cognitive behavioral therapies [11]. The specificity of this dimension has also gained support from genetic and functional imaging studies. The hoarding dimension has recently been used as a dichotomous and a quantitative trait in a genome wide scan in sib pairs with Tourette's syndrome [17]. Two specific loci on 5q and 4q could participate in the hoarding phenotype. Moreover, the left precentral gyrus and right orbitofrontal cortex are specifically activated during the provocation of hoarding-related anxiety in OCD [15].

### Symptom dimensions stability

Consistent with our initial hypothesis, the four factors that emerged from our baseline analysis in children and adolescents remained mainly unchanged across time. Within comparisons revealed that the symmetry/ordering, the obsessive, and the hoarding dimensions were unmodified across time. Only the contamination/cleaning dimension decreased, as previously reported by Mataix-Cols et al. [18] in adults. The reasons for the changes within the contamination/cleaning dimension but not in the other dimensions remain unclear. Certain dimensions could be more resistant to treatment [4,11], thus explaining why they remain unchanged across time. However, the contamination/cleaning dimension decreased independently of the reduction in OCD severity.

The naturalistic evolution of the disorder could partially explain these results. Indeed, the evolution of the disorder appears to be associated with a reduction in the multiplicity of OC symptoms independently of the severity of the disorder. This hypothesis may explain why Rettew et al. [6] and us observed a significant decrease in the number of obsession categories during follow-up, independently of the severity of the disorder. However, further studies, with a rigorous assessment of treatments used (cognitive behavior therapy and/or serotonergic inhibitors) and of clinical responses to treatments are needed.

Multiple regression analyses also reinforced the hypothesis that OC symptoms are stable over time. Shifts from one dimension to another were rare. The scores at the time of the reassessment of the symmetry/ordering, obsessive, and contamination/cleaning dimensions, were highly correlated to their scores at baseline. The hoarding dimension also showed a slight but non significant correlation between its scores at baseline and follow-up. However, the partial correlations that we obtained in our study (0.24 to 0.59) were weaker than those reported by Mataix-Cols et al. [18] in adults (0.40 to 0.86). Three main factors might explain this discrepancy. First, the size of our sample was limited, so the correlation coefficients we obtained could be less reliable than those estimated from larger samples. Second, the follow-up period was longer in our study than in that published by Mataix-Cols and co-workers (four years versus two years, respectively) and could thus explain why we observed a higher variability of symptom dimensions across time. Moreover, the re-evaluation of OC symptoms three times during the two years of follow-up in the study of Mataix-Cols et al. could have led to an overestimation of symptom stability induced by a rememoration bias. In our study, patients only re-fulfilled the questionnaire at the end of follow-up. Third, although our results showed that after follow-up the symptom dimensions identified remained essentially unmodified, about one third of children and adolescents in our sample showed modifications in most OC categories. By contrast, in the study of Mataix-Cols et al. [18], about 12.6 % of patients (6 to 22) showed modifications in symptom categories of the Y-BOCS after one to two years of follow-up. Thus, symptoms could be less stable in children than in adults when using both categorical and dimensional approaches.

Our results contrast with the marked variability of OC symptoms across time in children and adolescents initially reported by Rettew and co-workers [6]. These discrepancies are probably due to differences in the statistic methodology used in the two studies. Specifically, these authors concluded about that instability of OC symptoms studying symptom categories rather than symptom dimensions. Based on categories, we also observed that about one third of OCD children and adolescents in our sample showed modifications in most OC categories. Furthermore, it should be noted that although none of patients included in the report by Rettew et al. maintained the same constellation of symptoms across time, they found no significant differences in the rates of OC symptom categories that faded, emerged, or were maintained, whatever the categories. The authors only suggested that more changes could happen for religious, ordering, hoarding and somatic categories. This is in agreement with what we observed for religious and somatic obsessions in our sample. Finally, our patients reported many different symptoms spanning several categories, with a significant decrease in the number of obsessions across time but not of the compulsions, similarly to the findings reported by Rettew et al. [6]. Thus, when both studies are compared using similar statistic methodology, the results are not so divergent.

### Limitations

A number of methodological limitations in our study should be noted. First, the statistical methods used in this report to perform a factor analysis could bias the results. The Kaiser rule (eigenvalues >1) applied to determine the number of factors may lead to an overestimation of their number [29]. However, our study is the first to explore factor OC symptom structure in children and adolescents, and our results are in remarkable agreement with those of exploratory and confirmatory factor analytic studies in adults, consistently showing the emergence of the same four factors as the best-fit model.

Second, factor-analytic solutions are partly a function of the variables entered into the analysis. When different variables are entered, different factors may appear. The factorial structure obtained in our study largely depends on the major categories of the Y-BOCS, but exclude, as most of the studies in adults, miscellaneous obsessions and compulsions categories. The exclusion of these symptoms is not without consequence, since some of them are determinant in OCD phenotypic characterization. For instance, mental compulsions, which belong to the miscellaneous category, are among the most common compulsions in children [30], and are considered especially difficult to treat [31]. A recent study conducted in adults showed that miscellaneous symptoms could load nicely in the four main symptom dimensions [32]. Therefore, the dimensions that emerge from the factor analysis draw up a precise view of the major categories of the symptoms in the Y-BOCS, but a partial view of the symptomatic diversity of the disorder.

Third, one of the major limitations of our study is the restricted size of our sample. Factor analysis is very sensitive to the sizes of correlation, and correlation coefficients tend to be less reliable when estimated from small sample sizes [33]. It should be noted however, that the validity of factor analyses depends more on the loading and the number of the factors obtained than on the number of patients included. If the correlations are strong and a few, distinct factors are identified, small samples could be adequate. The factor loadings that we obtained in our study were in accordance with loading suggested by DeGeus and Denys [34], and the factor solution that we obtained was limited to four factors. In our sample of 73 patients included at baseline, the 95% confidence interval for an *r *value of 0.55 runs between 0.36 and 0.70. This kind of potential variability could greatly influence the factor structure. For instance, the counting compulsions, included in the second factor in our study (loading 0.56), could also be associated with the symmetry/ordering factor (loading 0.49) usually observed in previous reports.

Fourth, the follow-up period varied among patients from 1.3 to 5.3 years, so we do not know whether a more homogenous follow-up period would have given a different outcome. Fifth, the rate of patients that accepted to participate in the re-assessment was relatively low (57.5%) when compared, for example, to the report of Leonard et al. [35], where 89% of OCD patients were seen in person two to seven years after the initial assessment. These discrepancies could be due, in part, to the OCD severity of patients included at baseline. Leonard et al. [35] re-evaluated a sample of patients that were hospitalized and had a severe OCD at the time of baseline inclusion. Wewetzer et al. [36], who primarily included in- and out- patients as we did, reported similar difficulties to reassess juvenile OCD patients after follow-up, since only 49.5 % of their initial cohort was re-examined. The last limitation of our study concerns the inhomogeneous age of patients included in the study (7 to 18 years). We chose not to subdivide the patients in subgroups according to age because of the limited size of our sample. Additional investigations of symptom dimensions in large and homogenous groups of prepubescent children and adolescents are needed to further understand the course of OC symptoms in young patients.

## Conclusion

The present findings show for the first time that four symptom dimensions, similar to those previously described in adults and relatively stable overtime, underline the heterogeneity of OC symptoms in children and adolescents. The presence of clinical factors that are stable from childhood to adulthood strengthen the interest for investigating the clinical, neurobiological and genetic heterogeneity of OCD using a dimension-based approach. Working with subgroups defined according to dimensions may facilitate the identification of more specific etiopathogenic features underlying each of the dimension subgroups as well as factors implicated in the emergence of the disorder.

## Competing interests

The author(s) declare that they have no competing interests.

## Authors' contributions

RD conceived of the study, participated in data collection, interpreted the results, and drafted the manuscript. AB participated in the study design and data collection. CB participated in the interpretation of the results and in writing the manuscript. FM helped with the statistical analyses. NC helped to collect the data. ML was the principal investigator. All authors read and approved the final manuscript.

## Pre-publication history

The pre-publication history for this paper can be accessed here:


